# Suppression of thymosin β10 increases cell migration and metastasis of cholangiocarcinoma

**DOI:** 10.1186/1471-2407-13-430

**Published:** 2013-09-23

**Authors:** Sirinapa Sribenja, Kanlayanee Sawanyawisuth, Ratthaphol Kraiklang, Chaisiri Wongkham, Kulthida Vaeteewoottacharn, Sumalee Obchoei, Qizhi Yao, Sopit Wongkham, Changyi Chen

**Affiliations:** 1 Department of Biochemistry, Liver Fluke and Cholangiocarcinoma Research Center, Faculty of Medicine, Khon Kaen University, Khon Kaen, Thailand; 2 Molecular Surgeon Research Center, Division of Surgical Research, Michael E. DeBakey Department of Surgery, Baylor College of Medicine, Houston, TX, USA

**Keywords:** Thymosin β10, Cholangiocarcinoma, Cell migration, Cancer metastasis, Snail, ERK1/2, MMPs

## Abstract

**Background:**

Thymosin β10 (Tβ10) expression is associated with malignant phenotypes in many cancers. However, the role and mechanisms of Tβ10 in liver fluke-associated cholangiocarcinoma (CCA) are not fully understood. In this study, we investigated the expression of Tβ10 in CCA tumor tissues and cell lines as well as molecular mechanisms of Tβ10 in tumor metastasis of CCA cell lines.

**Methods:**

Tβ10 expression was determined by real time RT-PCR or immunocytochemistry. Tβ10 silence or overexpression in CCA cells was achieved using gene delivery techniques. Cell migration was assessed using modified Boyden chamber and wound healing assay. The effect of silencing Tβ10 on CCA tumor metastasis was determined in nude mice. Phosphorylation of ERK1/2 and the expression of EGR1, Snail and matrix metalloproteinases (MMPs) were studied.

**Results:**

Ten pairs of CCA tissues (primary and metastatic tumors) and 5 CCA cell lines were studied. With real time RT-PCR and immunostaining analysis, Tβ10 was highly expressed in primary tumors of CCA; while it was relatively low in the metastatic tumors. Five CCA cell lines showed differential expression levels of Tβ10. Silence of Tβ10 significantly increased cell migration, invasion and wound healing of CCA cells *in vitro*; reversely, overexpression of Tβ10 reduced cell migration compared with control cells (*P*<0.05). In addition, silence of Tβ10 in CCA cells increased liver metastasis in a nude mouse model of CCA implantation into the spleen. Furthermore, silence of Tβ10 activated ERK1/2 and increased the expression of Snail and MMPs in CCA cell lines. Ras-GTPase inhibitor, FPT inhibitor III, effectively blocked Tβ10 silence-associated ERK1/2 activation, Snail expression and cell migration.

**Conclusions:**

Low expression of Tβ10 is associated with metastatic phenotype of CCA *in vitro* and *in vivo*, which may be mediated by the activation of Ras, ERK1/2 and upregulation of Snail and MMPs. This study suggests a new molecular pathway of CCA pathogenesis and a novel strategy to treat or prevent CCA metastasis.

## Background

Cholangiocarcinoma (CCA), the malignancy of bile duct epithelial cells, is a major cancer and a main health problem in the northeast of Thailand [1,2]. A global increase in CCA related mortality and incidence of CCA have been reported [3,4]. Several conditions associated with chronic inflammation have been identified as risk factors for CCA. Infection with the liver fluke (*Opisthorchis viverrini*) is the major risk factor of CCA in Thailand and Southeast Asia [5]; whereas primary sclerosing cholangitis is the main risk factor in Western countries [6].

Since CCA is difficult to diagnose at an early stage, almost all patients with CCA present with advanced, incurable disease. Even in patients who have undergone complete surgical resection, the recurrence rate remains quite high and the 5-year survival rate is unfavorable [7,8]. CCA is a slow growing but highly metastatic cancer, which is the major cause of death in CCA patients. Currently, there are no effective chemotherapeutic drugs and sensitive tumor markers to diagnose or monitor the tumor progression; most of CCA patients present themselves with high metastasis to lymph nodes and blood vessels. Therefore, understanding the molecular mechanism underlying CCA metastasis will lead to development of new strategies for the diagnosis and the treatment of CCA.

We have established the serial analysis of gene expressions (SAGE) database of the primary and corresponding metastatic tumors from a Thai male patient with CCA, as well as high and low invasive CCA cell lines ( http://cgap.nci.nih.gov/SAGE). The differential expression of genes in primary vs. metastatic tumors has been recently reported [9]. Thymosin β10 (Tβ10) (TMSB10; SAGE tag: GGGGAAATCG) was highly expressed in primary CCA tumors; while it was reduced dramatically in the metastatic tumors (6.5 fold decrease). Furthermore, immunohistochemical (IHC) staining showed that the intensity of Tβ10 staining in the primary CCA tumor tissue was higher than that in the normal liver tissue. However, the impact of the suppression of Tβ10 on the metastasis of CCA is not known.

Tβ10 is a member of the β-thymosin family, which is widely distributed in many tissues with proven biological activities as an actin sequestering protein involved in cell motility. There are at least 15 β-thymosins discovered, of which Tβ4 and Tβ10 are the most commonly found in mammalian cells with Tβ4 being the major form (70 - 80%). Tβ4 and Tβ10 are mainly localized in cytoplasm, and have high affinity to G-actin (actin monomer); while the expression and functions of Tβ4 and Tβ10 are quite different [10-13]. Tβ10 is differentially expressed in embryogenesis and neuronal development. Its expression is also increased in many inflammatory conditions and tumorigenesis including cell proliferation, anti-apoptosis and angiogenesis [14-16]. However, the functional association of Tβ10 with tumor metastasis is controversial. High levels of Tβ10 expression were found in the metastatic tumor of thyroid [17,18] and cutaneous malignancy [19]; while the low level of Tβ10 expression was associated with metastatic cervical carcinoma [20] and CCA [9].

In this study, the expression of Tβ10 in the primary and metastatic CCA was determined. The functional role of Tβ10 in CCA cell migration and metastasis was studied in CCA cell lines and a nude mouse model of CCA xenograft. Moreover, the possible signaling pathway of Tβ10 in tumor metastasis was explored.

## Methods

### Patient tissues

Primary and corresponding metastatic CCA tissues (n = 10) were obtained from the specimen bank of the Liver Fluke and Cholangiocarcinoma Research Center. Specimens were collected from intrahepatic CCA patients who underwent surgery at Srinagarind hospital, Faculty of Medicine, Khon Kaen University. Informed consent was obtained from each subject before surgery, and the Human Research Ethics Committee at the Khon Kaen University, Thailand approved the research protocol. The specimens were kept frozen in Trizol (Invitrogen, CA) at -80°C until use.

### Cell lines and cell culture

CCA cell lines, KKU-M055, KKU-100, KKU-M156, KKU-M213 and KKU-M214, were established from primary tumors of Thai patients with different histological types [21,22]. All cell lines were cultured in the DMEM medium supplemented with 10% w/v fetal bovine serum (FBS), 100 U/mL penicillin and 100 μg/mL streptomycin at 37°C and 5% CO_2_.

### Chemicals and reagents

DMEM medium, fetal bovine serum (FBS), trypsin EDTA, Opti-MEM I medium and LipofectAmine™ 2000 transfection reagent were purchased from Invitrogen Life Technology (Grand Island, NY). Puromycin and mouse anti-β-actin antibody were purchased from Sigma Chemical Co (St Louis, MO). Rabbit anti-Tβ10 antibody was purchased from Biodesign International (Cincinnati, OH). Goat anti-rabbit IgG (H&L) antibody conjugated to horseradish peroxidase (HRP), goat anti-mouse IgG (H&L) antibody conjugated to HRP and rabbit anti-SNAl1 were obtained from Cell Signaling Technology Laboratories, Inc (Danvers, MA). Rabbit anti-ERK1/2 antibody, mouse anti-pERK1/2 antibody, mouse anti-Histone H1 and rabbit anti-EGR1 antibodies were obtained from Santa Cruz Biotechnology (Dallas, TX). The chemiluminescence (ECL) Prime Western Blotting Detection Reagent kit was purchased from GE Healthcare (Piscataway, NJ). The Ambion RNAqueous-4PCR kit and DNA removing kits were purchased from Ambion (Austin, TX). The iQ SYBR Green supermix and iScript cDNA synthesis kits were purchased from Bio-Rad (Hercules, CA). All other chemicals were from Sigma.

### RNA extraction

Total RNA was extracted using Ambion RNAqueous-4PCR kit following the manufacture’s instruction. Briefly, cells were lysed using lysis buffer, transferred to a mini-column and centrifuged at 10,000 × *g* for 1 min. The column was washed and eluted in 60 μL of elution buffer. RNA solution was treated with DNAse I to remove any trace amounts of genomic DNA contamination. The frozen mouse tumor tissues were soaked overnight in RNAlater-ICE buffer (Ambion) before RNA extraction.

### Real time RT-PCR

Tβ10 mRNA levels were determined using real time RT-PCR. Briefly, mRNA was reverse-transcribed into cDNA using the iScript cDNA synthesis kit and real time RT-PCR was performed using the iQ SYBR Green supermix kit (Bio-Rad, Hercules, CA). The PCR reaction of 100 nM of each primer, 20 ng cDNA templates and iQ SYBR Green supermix, ran for 40 cycles of 95°C for 20 sec and 60°C for 1 min. Each cDNA sample was run in duplicate. β-actin was used as an internal loading control. The mRNA levels of early growth response protein 1 (EGR1), Snail, MMP3, MMP7 and MMP9 were similarly determined. The relative mRNA level was presented as unit values of 2^[Ct(β-actin)–Ct(Tβ10)]^. The primers for human Tβ10 and β-actin were used as described in our previous publication [23].

### Immunocytochemistry

Cells were seeded into a 24-well plate (2x10^4^ cells/well) and incubated in 5% CO_2_ at 37°C for 24 h. Cells were fixed with 95% ethanol and washed twice in PBS, then exposed to 0.3% hydrogen peroxide in absolute methanol to quench endogenous peroxidase, and blocked with 5% FBS in PBS for 1 h. Cells were incubated with 1:500 rabbit anti-Tβ10 antibody (Biodesign, Cincinnati, OH) at 4°C overnight. To visualize antibody binding, cells were reacted with anti-rabbit IgG EnVision (Dako, Carpinteria, CA) for 30 min and diaminobenzidine (DAB) for 5 min. The reaction was stopped by washing with distilled water followed by Mayer’s haematoxylin staining.

### Nuclear extraction

Cells were collected and washed with PBS. Cells were lyzed in 1 mL hypotonic buffer (10 mM HEPES-KOH pH 7.9, 1.5 mM MgCl_2_, 10 mM KCl, 0.1% NP-40, 0.5 mM DTT and 1× Protease inhibitor cocktail) and incubated on ice for 15 min. Nuclei fraction was collected by centrifugation at 14,000 rpm for 30 sec, lyzed with 80 μL of nuclear lysis buffer (50 mM HEPES-KOH pH 7.9, 10% glycerol, 420 mM KCl, 5 mM MgCl_2_, 0.1 mM DTT and 1× Protease inhibitor cocktail), and incubated on ice for 30 min. Nuclear extracts were obtained by centrifugation at 14,000 rpm for 10 min.

### Western blot

Cells were lysed with radioimmuno-precipitation assay buffer (Pierce Biotechnology) for 30 min on ice. Whole cell lysates were then collected after centrifugation at 12,000 rpm for 10 min at 4°C. Whole cell and nuclear fraction lysate (30 μg) were loaded for ERK1/2, phosphorylated ERK1/2, EGR1 and Snail detection, respectively. Protein bands were separated with 12% Tris-Glycine SDS polyacrylamide gel electrophoresis and then transblotted for 2 h at 4°C onto Hybond-P PVDF membrane (GE Healthcare, Piscataway, NJ). The membrane was probed with rabbit anti-ERK1/2 antibody (1:2,000), mouse anti-pERK antibody (1:1,000) and anti-β-actin antibody (1:10,000) at room temperature for 1 h or rabbit anti-EGR1 (1:1000), rabbit anti-Snail (1:1000) and mouse anti-Histone H1(1:1000) antibody at 4°C overnight. Then, the membrane was incubated in a HRP-linked secondary antibody (1:20,000) for 1 h at room temperature; the immunoreactive bands were visualized using the chemiluminescence Prime Western Blotting Detection Reagent kit.

### Transient silence of Tβ10 by siRNA

KKU-M214 and KKU-100 CCA cells (with a high endogenous Tβ10 expression; 2x10^4^ cells/well) were seeded into a 6-well plate for 24 h before transfection. The siRNA specific sequence for targeting human Tβ10 (5′-GCGGAGUGAAAUUUCCUAA-3′), corresponding to nucleotides 199 to 217 in the human sequence, was obtained from Ambion (Austin, TX). The cells were transfected either with 50 pM siTβ10 or a control scramble RNA. Transfections were carried out by using the LipofectAmine™ 2000 (Invitrogen, CA) according to the manufacturer’s instructions. After siRNA transfection, the plates were incubated at 37°C for 24 h for further analysis and total RNA was isolated with Trizol (Invitrogen, CA) reagent and reverse transcription-PCR was done.

### Establishment of stable cell lines and single clone selection

To establish stable silence cell lines, shRNA plasmids and full-length cDNA plasmids used in the present study were purchased from OriGene and GeneCopoeia, respectively. Stable cells expressing Tβ10 shRNA were created in KKU-M055 and KKU-M214 cells by stably transfecting with HuSH 29mer shRNA construct against Tβ10 (sh-Tβ10) to elicit silencing by use of a retroviral delivery system (OriGene Rockville, MD), following manufacturer’s instructions. These were compared to cell lines transfected with the shRNA pRS non-effective GFP plasmids (sh-vector) as a negative control. The sequence of the Tβ10 shRNA used in this study is as follows: 5′-AGATGGACACGAGCCACAAGCTGCACTGT-3′. Briefly, Phoenix™ Ampho Cells (Origene, Rockville, MD) were transfected with either Tβ10 shRNA plasmid or shRNA vector control plasmid. Viral supernatants were collected and transduced into the parental KKU-M055 and KKU-M214 cells. Stable cell lines expressing Tβ10 shRNA (M055-sh-Tβ10 and M214-sh-Tβ10) or negative control vector (M055-sh-vector and M214-sh-vector) were selected with the addition of 1 μg/mL puromycin into the medium.

To generate stable overexpression stable cell lines by the lentiviral delivery system, full-length Tβ10 cDNA plasmid called pReceiver-Tβ10-Lv105 overexpression construct (GeneCopoeia) or pReceiver-eGFP-Lv105 vector as a control was co-transfected into 293T cells with HIV packing plasmids (GeneCopoeia). Viral supernatants were collected, filtered and transduced to the target cells. Stable cell lines expressing Tβ10 (M055-Lenti-Tβ10 and M213-Lenti-Tβ10) or GFP control (M055-Lenti-GFP and M213-Lenti-GFP) were selected with adding 0.5 μg/mL of puromycin into the medium. All stable cell lines were cultured for at least 2 weeks before use in experiments. Tβ10 expression was confirmed by real time RT-PCR analysis. Fluorescence images of cells were captured to observe GFP signal in GFP control cells.

For isolation of individual clones, the cells were grown in the complete culture medium and then digested into individual cells with 0.05% trypsin-EDTA and plated at a density of 500 cells per 100-mm cell culture dish in the presence of 0.5 μg/mL puromycin. Growth of the cell colonies was monitored by light microscopy. When the individual colonies reached approximately 100 - 200 cells, positions of the solitary colony were marked and single cell clones were isolated by sterile cloning cylinders. Selected 5-8 single cell clones were subjected to expansion culture until sufficient amounts of cells were obtained.

### *In vitro* migration

Cell migration was determined using a modified Boyden chamber assay. Uncoated- and pre-coated Matrigel-inserts (8 μm pore size Transwell®, Corning Inc., NY) were used for migration and invasion assay, respectively. Cells (1×10^5^) were seeded into the upper compartment of the chamber and 600 μL DMEM supplemented with 10% w/v FBS were placed into the lower chamber. After incubation at 37°C for an appropriate time, cells in the upper chamber were fixed with 4% w/v paraformaldehyde for 15 min and stained with 0.5% w/v crystal violet in 25% v/v methanol. Cells in the upper surface of the filter were scraped off using a cotton swab and the number of migrated cells in the lower surface was counted under microscope. Mean values of nine low-power fields (100× magnification) were determined. For stable cell lines, after cells migrated at 37°C for the specified time, the cells were incubated with Calcein-AM (Molecular Probes, Eugene, OR) for 1 h at 37°C before fixation. The fluorescence was read from the bottom at an excitation wavelength of 495 nm and emission wavelength of 520 nm. Cells in the upper chamber were then removed, and cells that had migrated onto the lower surface of the membrane were quantified. The migration rate was presented as the ratio of the mean fluorescence reading after scraping of the cells divided by the reading before removal of the top cells. Assays were done in triplicate and two independent experiments were repeated. In stable cell lines which incubated for migration more than 24 h, cells were pretreated with 12.5 ng/mL Mitomycin C (Sigma-Aldrich, St. Louis, MO) for 3 h before seeded on the upper chamber to inhibit cell proliferation.

### Monolayer cell wound healing

The stable cells were seeded into 6-well plates (1.5x10^6^ cells/well) and incubated in a humidified atmosphere of 5% CO_2_ at 37°C for 24 h. To inhibit cell proliferation, a potential confounding variable, all wound assay cells were pretreated with 5 μg/mL Mitomycin C for 3 h before the scrape line was made. Wounds were generated on the surface of confluent monolayers using a sterile pipette tip, followed by incubation with DMEM medium supplemented with 10% FBS. Healing was observed at different time points along the scrape line and a representative field for each cell line was photographed. Assays were done in triplicate and two independent experiments were repeated.

### Nude mouse model

The following animal work was approved by the Office for Protection from Research Risks and Animal Welfare Act guidelines under an animal protocol approved by the Baylor College of Medicine Institutional Animal Care and Use Committee. Subconfluent and stable M214-sh-Vector-GFP and M214-sh-Tβ10-GFP cells were harvested and resuspended in serum-free DMEM. For intrasplenic injection, mice were anesthetized with 2.5% avertin, and a 0.5- to 1-cm incision was made in the left subcostal region. The spleen was exteriorized and the tumor cells (2 × 10^6^ cells) in a volume of 50 μL were injected into the tail of spleen of 5 to 6-week-old male nude mice (NCI Charles River); four animals per group were used. The peritoneum and skin were closed with a 4.0 surgical suture. Mouse body weight was measured weekly. After 20 days of tumor implantation, all mice were euthanized by an overdose of 2.5% avertin and evaluated macroscopically for the presence of primary tumors in the spleen and the metastases in the liver, lung and abdominal cavity. To observe the gross nodule of liver metastasis, the whole livers were imaged under a fiber optic illumination LT-9900 Illumatool Bright Light System (Lightools Research, Encinitas, CA), and imaging was carried out at 470 nm with LT-9470FX 470 nm in Lightools Filter Cup (Lightools Research, Encinitas, CA). The total number of green fluorescent protein (GFP)-positive nodules in the surface of all lobes of liver was quantified. Next, the primary tumor site (spleen) and other organs (liver and lungs) were harvested; the whole organ of each specimen was embedded in Tissue-Tek® OCT compound (Sakura Finetek Inc., Torrance, CA) and snap-frozen in liquid nitrogen before storage in -80°C for further histological studies. For the micrometastasis study, liver dissections were sampled from the caudate and left lobe of each mouse. 14-μm frozen sections were cut in a cryostat by following cryostat manufacturer’s recommendation; then, the slides were fixed in 4% paraformaldehyde for 10 min at room temperature. All were visualized under an inverted fluorescent microscope using a 10× objective to verify the green signal of GFP. Six fields of GFP indicated areas of each liver section were taken and the number of micrometastasis was quantified. Tumors inside the abdominal cavity were stored in RNAlater solution (Ambion, Austin, TX) for real time RT-PCR analysis.

### Statistical analysis

Experimental data were analyzed using SPSS 13.0 Windows Evaluation software (SPSS Inc., Chicago, IL). All quantitative data were expressed as mean or percentage ± SD. Two-tailed Student’s *t*-test was used for comparison between two groups. Statistical significance was established at *P* < 0.05.

## Results

### Tβ10 expression is decreased in the metastatic tumor of liver fluke-induced cholangiocarcinoma

**Figure 1:**
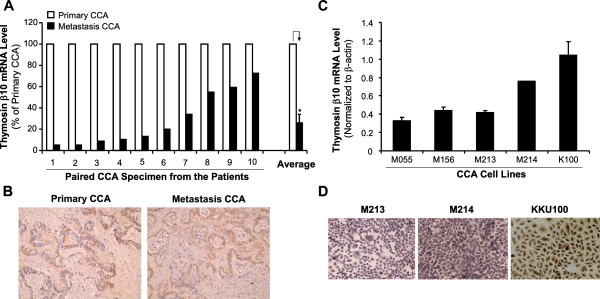
**Endogenous expression of Tβ10 in CCA tissue specimens and cell lines.** To investigate the expression of Tβ10 in CCA, 10 pairs of CCA surgical specimens were collected from the operating room under an approved research protocol. **(A)** Tβ10 mRNA levels of the primary tumor and metastatic tumor of each CCA case were analyzed by using real time RT-PCR. Tβ10 mRNA levels were significantly lower in the metastatic site than that in the primary tumor tissue (*P<0.05; n=10). **(B)** Tβ10 protein levels of a representative CCA case of paired tissues were determined by immunohistochemistry. **(C)** Tβ10 mRNA levels were also examined in 5 CCA cell lines (KKU-M055, KKU-M156, KKU-M213, KKU-M214 and KKU-100) by real time RT-PCR. **(D)** Tβ10 protein levels were determined by immunocytochemistry in three CCA cell lines (KKU-M213, KKU-M214 and KKU-100).

Our SAGE data indicates that Tβ10 may play a role in CCA metastasis [9]. In this study, we determined the expression of Tβ10 in 10 pairs of CCA surgical specimens (primary and metastatic tumor) and 5 CCA cell lines previously isolated from the CCA tissues [21,22]. High expression of Tβ10 was found in the primary CCA tumor; while significantly low expression of Tβ10 was observed in the metastatic tumor by real time RT-PCR analysis and immunohistochemistry staining (Figure 1A, 1B). We also observed different endogenous Tβ10 levels among 5 CCA cell lines (Figure 1C, 1D). Three CCA cell lines (KKU-M055, KKU-M156 and KKU-M213) had a relatively low expression of Tβ10; while other two cell lines (KKU-M214 and KKU-100) had a relatively high expression of Tβ10. These expression data provide a strong rationale for the functional analysis of Tβ10 in CCA.

### Silence of Tβ10 promotes cell migration and monolayer wound healing in liver fluke-induced cholangiocarcinoma cells

**Figure 2:**
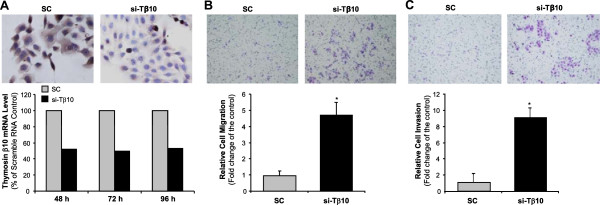
**Effects of transient silence of Tβ10 on cell migration in KKU-M214 cell line. (A)** KKU-M214 cells were treated with 50 pM Tβ10 siRNA or scramble siRNA as a control for 48, 72 and 96 h, respectively. Tβ10 mRNA and protein levels were determined by real time RT-PCR and immunohistochemistry, respectively. **(B)** Migration and **(C)** invasion assays were carried out in KKU-M214 cells using a modified Boyden chamber assay. Cells were pre-treated with Tβ10 siRNA or scramble siRNA for 48 h, and placed on Transwell to determine cell migration (15 h) and on Transwell with pre-coated Matrigel (18 h) to determine cell invasion. Bar graphs represent fold change. n = 3, *P<0.05 versus the control.

To study the potential function of Tβ10 in CCA, we determined the effect of Tβ10 silence on cell migration in a KKU-M214 cell line, which showed a high expression of Tβ10. KKU-M214 cells were transfected with 50 pM of Tβ10 siRNA (Ambion); and this reduced Tβ10 mRNA levels by 50% at different time points (48, 72 and 96 h) and Tβ10 protein levels dramatically by immunocytochemistry analysis (Figure 2A). We performed migration and invasion assays by using a modified Boyden chamber method, and found that silence of Tβ10 significantly enhanced cell migration and invasion of KKU-M214-siTβ10 cells at 15 h and 18 h, compared with those of KKU-M214-scramble RNA cells transfected with the scramble RNA (Figure 2B, 2C; *P<0.05; n = 3).

**Figure 3:**
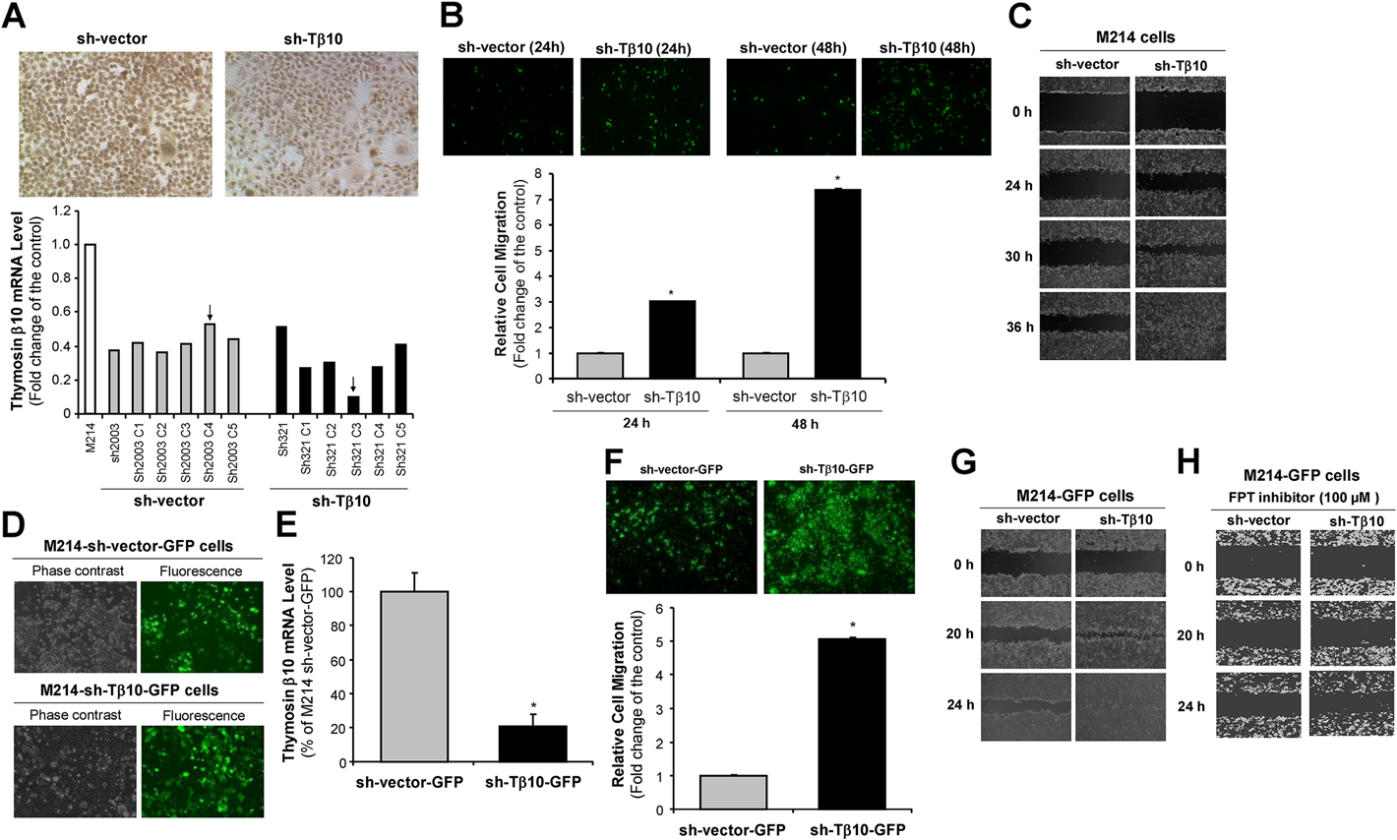
**Effects of stable silence of Tβ10 on cell migration and monolayer wound healing in KKU-M214 cell line.** Stable cell lines expressing small hairpin Tβ10 RNA (sh-Tβ10) or control vector (sh-vector) were generated in KKU-M214. **(A)** All clones of control and Tβ10 silencing cells were confirmed by real time RT-PCR. β-actin was used as an internal control. Arrows indicate selected clone for use in subsequent experiments; and insert pictures show immunocytochemistry results of selected clones. **(B)** Stable Tβ10 silence led to enhanced KKU-M214 cell migration *in vitro* in 24 and 48 h incubation by the modified Boyden chamber assay. Bar graphs represent fold change. n = 3; *P<0.05 versus the control. **(C)** Wound healing assay was carried out in M214-sh-vector and M214-sh-Tβ10 cells using DMEM medium supplemented with 10% FBS. Representative images were taken from the same field at 0, 24, 30 and 36 h. **(D)** M214-sh-vector-GFP and M214-sh-Tβ10-GFP cells were established, showing GFP signals. **(E)** The expression levels of Tβ10 in stable silence cells (M214-sh-Tβ10-GFP cells and M214-sh-vector-GFP cells) were determined by real time RT-PCR. n = 3, *P<0.05 versus the control. **(F)** Migration assay was carried out with M214-sh-vector-GFP and M214-sh-Tβ10-GFP cells at 48 h incubation by the modified Boyden chamber assay. n = 3, *P<0.05 versus the control. **(G)** Wound healing assay for M214-sh-vector-GFP and M214-sh-Tβ10-GFP cells. Representative images were taken from the same field at 0, 20, and 24 h. **(H)** M214-sh-vector-GFP and M214-sh-Tβ10-GFP cells were pre-treated with a Ras-GTPase inhibitor (FPT inhibitor III) for 2 h. Wound healing assay was performed. Representative images were taken from the same field at 0, 20, and 24 h.

To further confirm the role of Tβ10 silence in CCA migration, we established stable cell lines with Tβ10 silence in two CCA cell lines KKU-M214 and KKU-M055 by the retroviral vector delivery system and puromycin selection. Silencing of Tβ10 in these cell lines was carefully confirmed by real time RT-PCR. The Tβ10 mRNA level of all single clones of M214-sh-Tβ10 cells and scramble vector control cells as well as a representative of Tβ10 immunoreactivity are shown in Figure 3A. For the cell migration assay, silence of Tβ10 in M214-sh-Tβ10 cells was associated with 2.5 to 3-fold increase in cell migration at 24 h and 48 h, respectively, compared with that in M214-sh-vector cells (Figure 3B, *P<0.05; n = 3). Similar results were also obtained in the monolayer wound healing assay; and low expression of Tβ10 resulted in an increase in cell migration of M214-sh-Tβ10 cells compared with that of M214-sh-vector cells in the presence of 5 μg/mL Mitomycin C, which inhibits cell proliferation (Figure 3C).

In a parallel experiment, the M214-sh-vector and M214-sh-Tβ10 cells were established by double transfection with an eGFP expressing vector for use as a reporter signal for the imaging purpose in the animal study. To ensure that addition of eGFP did not alter Tβ10’s function in these cells, we performed the migration and wound healing assay in Tβ10 stable knockdown cells (M214-sh-Tβ10-GFP). Cells infected with Lentivirus contained-eGFP plasmid were selected in 1 μg/mL puromycin for 1 week before use in experiments. Phase contrast images of cells were captured on an inverted fluorescent microscope using a 10× objective to verify the green signal of GFP (Figure 3D). After transducing eGFP into the cells, we confirmed the expression of Tβ10 in M214-sh-vector-GFP and M214-sh-Tβ10-GFP cells (Figure 3E). Then, we determined the effects of Tβ10 silence once again. Indeed, Tβ10 silence significantly increased the cell migration and monolayer wound healing in M214-sh-Tβ10-GFP cells compared with those in M214-sh-vector-GFP cells (Figure 3F, 3G). eGFP did not affect the function of Tβ10 silence *in vitro*.

**Figure 4:**
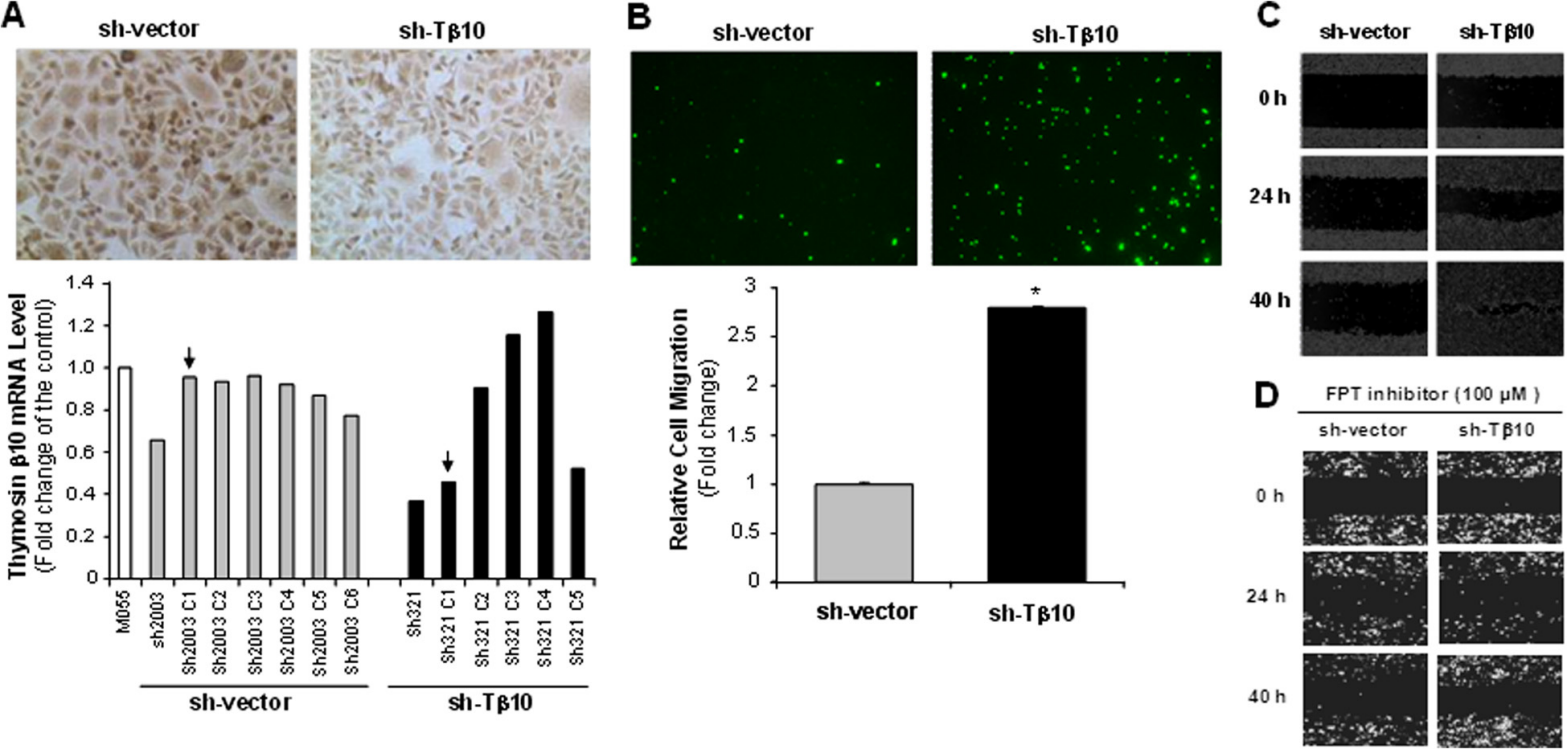
**Effects of stable silence of Tβ10 on cell migration and monolayer wound healing in KKU-M055 cell line.** Stable cell lines expressing Tβ10 shRNA and control vector (sh-vector) were generated in KKU-M055 cell line. **(A)** All clones of control and Tβ10 silencing cells were confirmed by real-time RT-PCR. β-actin was used as an internal control. Arrows indicate selected clone for use in subsequent experiments; and insert pictures show immunocytochemistry results of selected clones. **(B)** Cell migration of sh-Tβ10 and sh-vector cells was studied by the modified Boyden chamber assay at 24 h incubation. Bar graphs represent fold change or percentage of control. n = 3; *P<0.05 versus the control. M055 sh-vector and M055 sh-Tβ10 cells did not express eGFP gene. The green fluorescent signal in these cells was from Calcein-AM staining for the migration assay. **(C)** Wound healing assay was determined in sh-Tβ10 and sh-vector cells. Representative images were taken from the same field at 0, 24 and 40 h. **(D)** M055-sh-vector-GFP and M055-sh-Tβ10-GFP cells were pre-treated with a Ras-GTPase inhibitor (FPT inhibitor III) for 2 h. Wound healing assay was performed. Representative images were taken from the same field at 0, 20, and 24 h.

The functional role of Tβ10 silence was confirmed in another CCA cell line KKU-M055, which has a relatively low expression of Tβ10 (about 50% of Tβ10 in M214). More CCA cell types studied in this project could demonstrate that the effect of Tβ10 silence on CCA migration is not cell type specific. KKU-M055 was chosen for both knockdown and overexpression of Tβ10. Stable silence of Tβ10 was successfully established in M055 (Figure 4A); silence of Tβ10 was associated with 3-fold increased cell migration at 24 h in M055-sh-Tβ10 cells, compared with those in sh-vector control cells (Figure 4B, *P<0.05; n = 3). For the monolayer wound healing assay, one directional migration was substantially increased in M055-sh-Tβ10 cells, compared with that in M055-sh-vector control cells (Figure 4C). These results demonstrate that Tβ10 negatively regulates CCA cell migration *in vitro*, which may play a critical role in the metastasis of CCA.

### Forced overexpression of Tβ10 decreases cell migration and monolayer wound healing in fluke-induced cholangiocarcinoma cells

**Figure 5:**
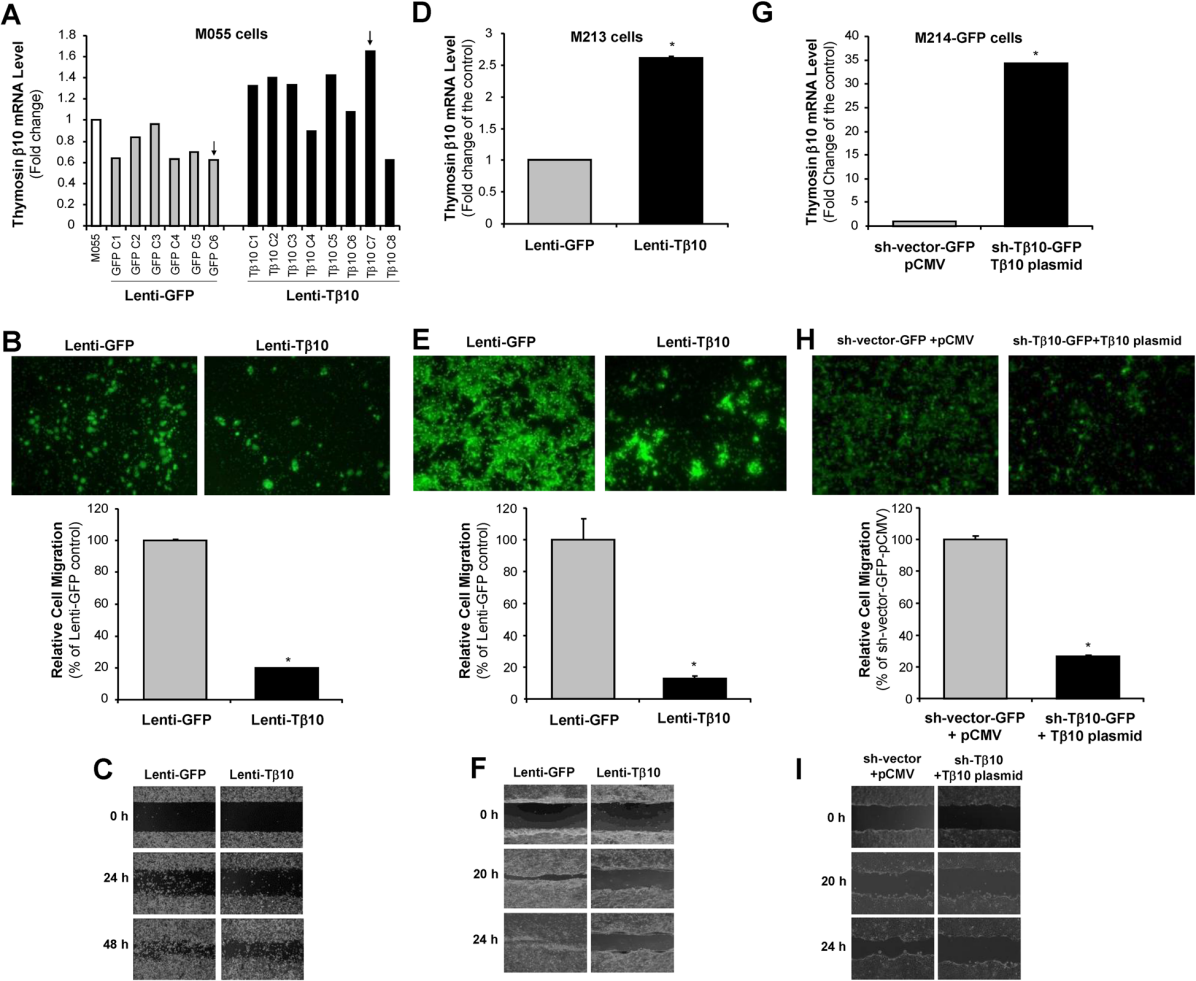
**Effects of stable overexpression of Tβ10 on cell migration and monolayer wound healing in KKU-M055, KKU-M213 and KKU-M214 sh-T**β**10-GFP cells.** Stable cell lines overexpressing Tβ10 (Lenti-Tβ10) and GFP control plasmid (Lenti-GFP) were generated in KKU-M055 and KKU-M213 cell lines. Selected clones of control and Tβ10 overexpressing KKU-M055 **(A)** and KKU-M213 **(D)** cells were confirmed by real time RT-PCR. β-actin was used as an internal control. Arrows indicate selected clone for use in subsequence experiments. Migration potential of KKU-M055 **(B)** and KKU-M213 **(E)** cells expressing Lenti-Tβ10 and its controls was determined by the modified Boyden chamber assay at 36 h incubation. Bar graphs represent fold change or percentage of control. n = 3; *P<0.05 versus the control. Wound healing assay was determined in KKU-M055 **(C)** and KKU-M213 **(F)**. Representative images were taken from the same field at 0, 24, and 48 h in KKU-M055, and 0, 20, and 24 h in KKU-M213 overexpression stable cell line. **(G)** Rescue experiment in KKU-M214 sh-Tβ10-GFP cells. KKU-M214 sh-Tβ10-GFP cells were transfected with Tβ10 expressing plasmid for 48 h; while the sh-vector-GFP cells received pCMV vector plasmid as a control. The expression levels of Tβ10 in these cells were determined by real time RT-PCR. **(H)** Cell migration of M214 sh-Tβ10-GFP + Tβ10 plasmid cells and sh-vector-GFP + pCMV cells were determined by the modified Boyden chamber assay at 60 h incubation. Bar graphs represent percentage of control. n = 3; *P<0.05 versus the control. **(I)** Wound healing assay was determined in M214 sh-Tβ10-GFP + Tβ10 plasmid cells and sh-vector-GFP + pCMV cells. Representative images were taken from the same field at 0, 20, and 24 h.

In order to further confirm the critical functions of Tβ10 in cell migration, we determined the effects of Tβ10 overexpression in two CCA cell lines KKU-M055 and KKU-M213, which have a relatively low endogenous level of Tβ10. The stable cell lines were established by a lentiviral vector delivery system including pReceiver-Tβ10-Lv105 overexpression construct and pReceiver-eGFP-Lv105 control vector (GeneCopoeia). By real time RT-PCR analysis, overexpression of Tβ10 in KKU-M055 (M055-Lenti-Tβ10) or KKU-M213 (M213-Lenti-Tβ10) cell lines was confirmed, compared with the M055-Lenti-GFP or M213-Lenti-GFP control cells, respectively (Figure 5A, 5D). In Figure 5A, we chose the M055 control cell clone (GFP C6), which had a lowest expression of Tβ10, and the M055 stable overexpression clone (Tβ10 C7), which had a highest expression of Tβ10, for further study because these clones may be more sensitive to determine the function of Tβ10 in CCA. Tβ10 mRNA levels in M055-Lenti-Tβ10 cells or M213-Lenti-Tβ10 cells were increased by 2.7-fold or 2.5-fold, respectively, compared with M055-Lenti-GFP cells or M213-Lenti-GFP cells. For the cell migration assay, cell migration of M055-Lenti-Tβ10 cells or M213-Lenti-Tβ10 cells was 80% or 87% lower than those of M055-Lenti-GFP cells or M213-Lenti-GFP cells at 36 h, respectively (Figure 5B, 5E, *P<0.05; n = 3). For the monolayer wound healing assay, Tβ10 forced overexpression also resulted in a lower cell migration rate in both M055-Lenti-Tβ10 and M213-Lenti-Tβ10 cells, compared with that in their vector control cells (Figure 5C, 5F). These data demonstrate the suppression role of Tβ10 in cell migration of CCA.

To determine the specificity of the functional role of Tβ10 in CCA, we performed a rescue experiment in M214 sh-Tβ10-GFP cells, which have a reduced Tβ10 level and increased cell migration. We hypothesized that reintroducing Tβ10 into this cell line would reverse its phenotype. We transiently transfected a pCMV6-XL5-Tβ10 overexpression plasmid (OriGene) into the M214 sh-Tβ10-GFP cells and found that their Tβ10 expression was 35-fold greater than those of pCMV6-XL5 empty vector control cells (Figure 5G). More importantly, forced Tβ10 overexpression completely reversed the promotion of cell migration caused by shRNA silencing of Tβ10 in both the modified Boyden chamber and the monolayer wound healing assays (Figure 5H, 5I).

### Stable silence of Tβ10 promotes tumor metastasis of fluke-induced cholangiocarcinoma cells in nude mice

**Figure 6:**
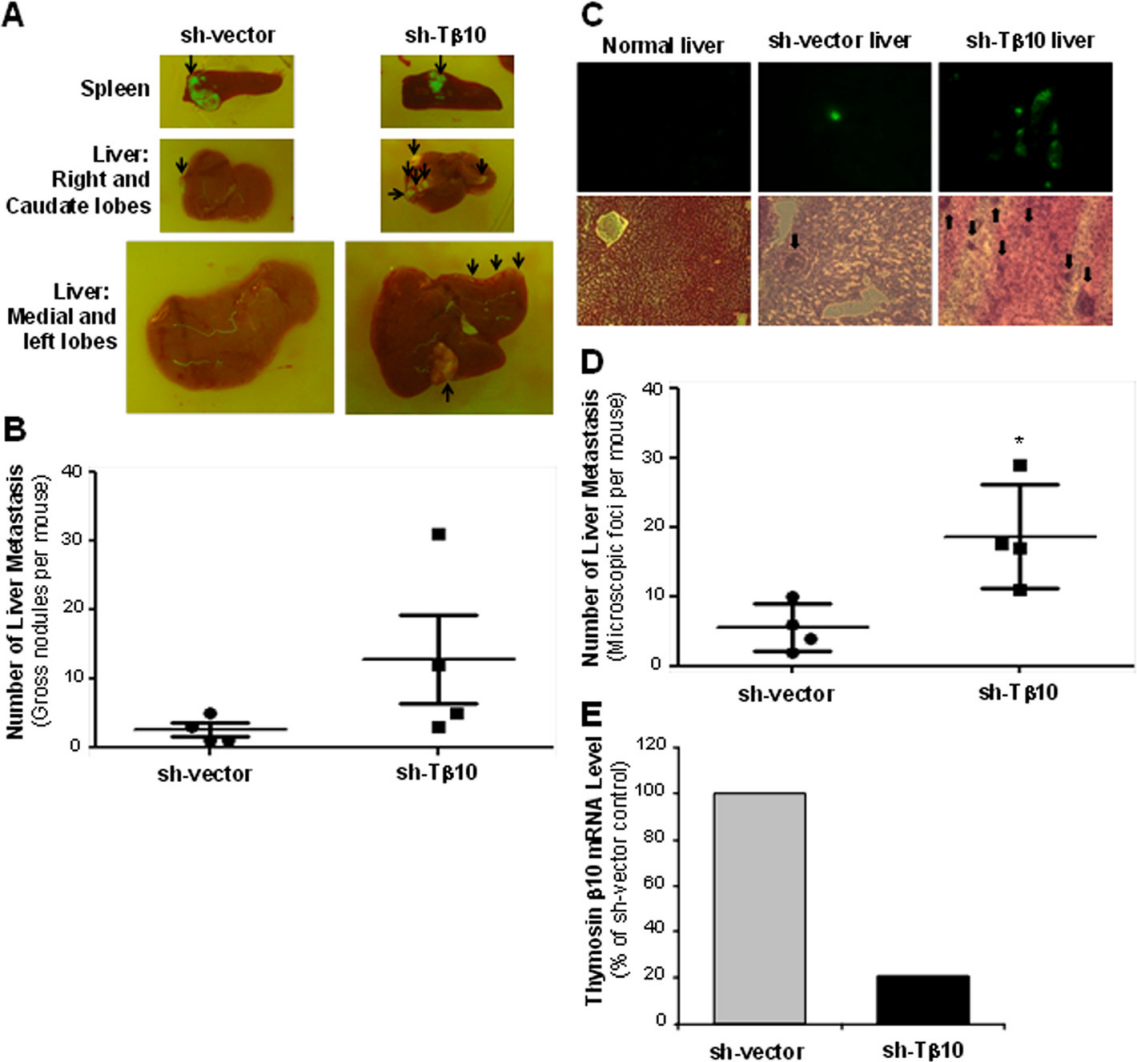
**Effects of stable silence of Tβ10 on tumor metastasis of KKU-M214 cell line in nude mice. (A)** M214-sh-vector-GFP cells or M214-sh-Tβ10-GFP cells were injected orthotopically into the spleen of nude mice for 3 weeks. Spleen (top), right and caudate lobes of liver (middle), medial and left lobes (bottom) were excised and GFP expressing tumors (black arrows) were examined using an UV illuminating system. **(B)** Graph showing the total number of GFP+ gross liver nodules in individual livers (±SD; n = 4). **(C)** Representative pictures of liver micrometastasis. Frozen sections of the liver were cut from the caudate and left lobe of each mouse and visualized under a inverted fluorescent microscope using a 10× objective to verified the green signal of GFP; and 6 fields of GFP indicated area of each liver section were taken (top) and stained for H&E (bottom). **(D)** The number of liver micrometastasis foci was quantified (±SD; n = 4, *P<0.05 versus the control). **(E)** Tβ10 silence persisted in the M214 sh-Tβ10-GFP cell line-derived tumors compared with M214 sh-vector-GFP cell line-derived tumors by real-time RT-PCR.

As shown in Figure 3D, we established M214 sh-Tβ10 and M214 sh-vector control cells with GFP expression, which can be used for the imaging of tumor metastasis *in vivo*. The effect of Tβ10 silence on the metastasis of CCA was analyzed *in vivo* using an immunodeficient nude mouse model. Twenty days after cells were injected orthotopically into the spleen of nude mice, the mice were sacrificed, and liver metastases were examined. The number of tumor metastasis nodules of the liver in the group of the mice injected with M214 sh-Tβ10-GFP cells was greater than that in the mice injected with M214 sh-vector-GFP control cells (Figure 6A, 6B). In addition, metastasis nodules were observed in omental parenchyma in 3 out of 4 mice injected with M214 sh-Tβ10-GFP cells; while 1 out of 4 mice injected with M214 sh-vector-GFP control cells had metastasis in the omental parenchyma. To observe liver micrometastasis, the liver tissues were sectioned and imaged for fluorescent GFP signal (CCA tumors), and the number of liver micrometastatic foci was counted under the fluorescent microscope. Micrometastatic lesions in the livers of mice injected with M214 sh-Tβ10-GFP cells were significantly more than that of mice injected with M214 sh-vector-GFP control cells (Figure 6C, 6D, *P<0.05, n = 4). We confirmed that Tβ10 silence persisted in the nude mouse tumor derived from M214 sh-Tβ10-GFP cells by real-time RT-PCR (Figure 6E). These results demonstrate that stable silence of Tβ10 promotes the liver metastasis of CCA cells in the nude mouse model.

### Silence of Tβ10 activates signaling pathways involved in tumor metastasis in fluke-induced cholangiocarcinoma cells

**Figure 7:**
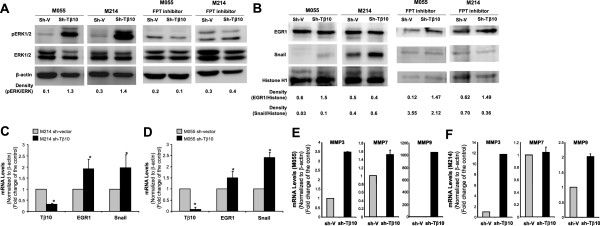
**Effects of Tβ10 silence on ERK1/2 phosphorylation and the expression of EGR1, Snail and MMPs in KKU-M055 and KKU-M214 cell lines. (A)** M055 sh-Tβ10 cells and M214 sh-Tβ10 cells as well as their vector control cells were grown in the complete medium for 24 h. Phosphorylated form and total form of ERK1/2 were determined by Western blot analysis. β-actin was used as a loading control. Intensity of the immunoreactive bands was measured by ImageJ software. This assay was repeated with pretreatment of a Ras-GTPase inhibitor (FPT inhibitor III, 100 μM, 2 h). **(B)** EGR1 and Snail protein levels in M055 sh-Tβ10 cells and M214 sh-Tβ10 cells as well as their vector control cells were determined by Western blot. Histone H1 was also included as a loading control. Intensity of the immunoreactive bands was measured by ImageJ software. This assay was repeated with pretreatment of a Ras-GTPase inhibitor (FPT inhibitor III, 100 μM, 2 h). The mRNA levels of Tβ10, EGR1 and Snail in M055 sh-Tβ10 cells **(C)** and M214 sh-Tβ10 cells **(D)** as well as their vector control cells were determined by real time RT-PCR analysis. Also, the mRNA levels of MMP3, MMP7 and MMP9 in M055 sh-Tβ10 cells **(E)** and M214 sh-Tβ10 cells **(F)** as well as their vector control cells were determined by real time RT-PCR analysis. All data were normalized to β-actin mRNA levels in these cells.

It is well known that ERK1/2, EGR1 and the zinc-finger transcription factor, Snail, play critical roles in tumor metastasis in several cancer types [24-27]. However, it is not clear whether these signaling pathways are involved in the CCA. Cell lysates and nuclear extracts from KKU-M055 and KKU-M214 cell lines with stable Tβ10 silence or vector control cells were harvested and used for immunoblotting to detect the levels of total and phosphorylated ERK1/2, EGR1 and Snail. β-actin and histone H1 were used for loading controls. Stable silence of Tβ10 in both KKU-M055 and KKU-M214 substantially activated ERK1/2 and increased Snail protein levels, but not EGR1 protein levels compared with that in vector control cells (Figure 7A, 7B). The mRNA levels of Snail and EGR1 were substantially increased in these CCA cells with Tβ10 silence (Figure 7C, 7D). Thus, ERK1/2 and Snail pathways may be involved in the functional role of Tβ10 silence-induced metastasis in CCA.

Since activated Ras can stimulate ERK1/2 in many cancer types [28], we hypothesized that the Ras-GTPase inhibitor may block activation of ERK1/2 and expression of EGR1 and Snail in Tβ10-silenced CCA cell lines. We treated stable Tβ10 knockdown cells (M055-sh-Tβ10 and M214-sh-Tβ10) and their vector control cells (M055-sh-vector and M214-sh-vector) with a Ras-GTPase inhibitor, FPT inhibitor III, (100 μM, Calbiochem, San Diego, CA) and performed Western blot analysis for phosphorylation of ERK1/2 and expression of EGR1 and Snail protein. FPT inhibitor III significantly inhibited activation of ERK/1/2 in both M055-sh-Tβ10 and M214-sh-Tβ10 cells (Figure 7A); and FPT inhibitor III also inhibited upregulation of Snail in both M055-sh-Tβ10 and M214-sh-Tβ10 cells (Figure 7B). In addition, pretreatment of FPT inhibitor III (100 μM for 2 h) inhibited the wound healing in both M214-sh-Tβ10-GPF cells (Figure 3H) and M055-sh-Tβ10 cells (Figure 4D).

Matrix metalloproteinases (MMPs) also play a critical role in cancer migration, invasion and metastasis [29]. We determined the expression of MMP3, MMP7 and MMP9 in stable Tβ10 knockdown cells (M055-sh-Tβ10 and M214-sh-Tβ10) and their vector control cells (M055-sh-vector and M214-sh-vector) by real time RT-PCR analysis. M055-sh-Tβ10 cells had a higher mRNA level of MMP3, MMP7 and MMP9 than the vector control cells had (Figure 7E). Similarly, M214-sh-Tβ10 cells had a higher expression of MMP3 and MMP9 than M214-sh-vector cells had (Figure 7F). Thus, the loss of Tβ10 in CCA may increase the expression of MMPs, which contribute to the enhanced migration and invasion of CCA cells.

## Discussions

In the current study, the functional role of Tβ10 in cell migration and tumor metastasis of CCA cell lines were investigated. Suppression of Tβ10 expression in CCA cell lines using siRNA-Tβ10 or shRNA-Tβ10 increases cell migration *in vitro* and enhances tumor metastasis in the nude mouse model. These results strongly suggest that suppression of Tβ10 in the primary CCA may increase its aggressiveness, possibly triggering some key signaling pathways for tumor metastasis.

There are numerous studies suggesting the critical roles for Tβ10 in tumorigenesis and progression of human cancers [20,23,30-34]. Expression of Tβ10 has been shown to confer cell migratory advantage in thyroid carcinoma [17,18,35,36], and melanoma [19,31,37]; but disadvantage in endothelial cells [38] and ovarian cancer [24]. However, roles of Tβ10 in cancer development such as cell growth and apoptosis still remain controversial among cancers [15,16]. At present, little is known about the expression and functions of Tβ10 in CCA. Using expressed sequence tags, Tβ10 was reported to be upregulated in intrahepatic CCA compared with normal liver tissues [39]. In this study, however, using real-time RT-PCR, we provide evidence, for the first time, that Tβ10 is upregulated in primary CCA; while it is significantly decreased in the metastatic CCA tumors. Functionally, reducing Tβ10 expression by transiently and stably silencing technologies significantly enhanced the migration of CCA cell lines.

Recently, there have been many reports that describe the potential functional roles of Tβ10 in human cancers; however, these functions are quite different among different types of cancers. Tβ10 induces antiproliferative and proapoptotic effects in ovarian cancer; while in pancreatic cancer, Tβ10 stimulates secretion of proinflammatory cytokines interleukin (IL-7) and IL-8, which may promote pancreatic cancer pathogenesis and progression [23]. Tβ10 inhibits tumor growth, angiogenesis, migration, and invasion of ovarian cancer *in vitro* and *in vivo* studies by disrupting actin polymerization and by inhibiting Ras action [24]. In our study, we demonstrate that Tβ10 silence significantly promotes cell migration in CCA cell lines (KKU-M055 and KKU-M214 cells); while forced overexpression of Tβ10 in CCA cell lines (KKU-M055, KKU-M213) has an inhibitory effect on CCA migration. The function of Tβ10 is specific because the effect of Tβ10 silence can be reversed by overexpression of Tβ10 in CCA cell lines. Tβ10 transiently silenced by siRNA oligonucleodie in KKU-M214 cells significantly increased both migration and invasion in M214 cells *in vitro*. However, the invasion was increased more than the migration in M214 cells with Tβ10 silence. The reason for the difference of invasion and migration in the same cell type is not clear. It is possible that the migration and invasion have different molecular mechanisms. Invasion requires local proteolysis of the extracellular matrix (ECM), pseudopodial extension, and cell migration [40,41].

From technical aspects, sh-RNA retrovirus construct for Tβ10 (sh-Tβ10) and empty control vector (sh2003) were used to infect both M214 and M055 CCA cells to establish stable silence cell lines by puromycin selection. Control vector nonspecifically reduced Tβ10 mRNA in M214 clones, but did not affect Tβ10 levels in M055. It is possible that different types of cells may contribute to this discrepancy. M214 was derived from a moderately differentiated CCA; while M055 was derived from a poorly differentiated CCA [21,22]. For the wound healing assay, control cells M055 Lenti-GFP had a lower wound healing rate compared with the control cells M213 Lenti-GFP although both cell types had a similar expression level of Tβ10. It is possible that different types of CCA cell lines have different mechanisms to control cell migration. Under the culture condition, M055 cells grow slower than M213 cells. In the rescue experiment, Tβ10-overexpressing plasmid was transiently transfected into the Tβ10 stable knockdown cells (M214 sh-Tβ10-GFP) and caused a 35-fold increase of Tβ10 mRNA levels compared with that in vector control cells. It is possible that the overexpression of Tβ10 from the transiently transfected plasmid was strong and overcome sh-Tβ10-mediated degradation of Tβ10 in these rescue cells.

More importantly, we also demonstrate that silence of Tβ10 in CCA cell lines enhanced tumor metastasis in the nude mouse model. These data may indicate clinical significance of the suppression of Tβ10 in metastatic CCA. Our results were consistent with previous studies in endothelial cells [38] and ovarian cancer [24,42,43].

However, it is not clear why metastatic CCA has a reduced expression of Tβ10. A current study has reported that approximately 16.7% of CCA have *KRAS* mutations [44], resulting in constitutively active Ras, which may contribute to the loss of Tβ10 expression. Other studies report that Tβ10 is differentially regulated by many factors such as retinoic acid and retinoids, growth factors and steroid hormones. For examples, vascular endothelial growth factor (VEGF), thyroid-stimulating hormones (TSH) upregulate Tβ10 expression in a dose-dependent manner [15,16]. Moreover, chemotherapeutic drugs such as 5-Fluorouracil (5-FU) has been shown to affect Tβ10 expression [45]. Thus, Tβ10 could be an important biomarker for 5-FU treatment.

Cell migration is a complex biological process involving highly orchestrated multistep process network of proteins and regulatory pathways. One of these regulatory pathways is the ERK1/2 MAPK pathway, which transduces extracellular signals into intracellular responses and is necessary for many cellular events [46,47]. To address regulatory pathways, which are associated with the functional role of Tβ10 silence in CCA, we determined the correlation between Tβ10 silence and activation of ERK1/2. Indeed, when Tβ10 was silenced in CCA cell lines, phosphorylation of ERK1/2 was substantially increased. It has been reported that ERK-mediated phosphorylation of FAK at Ser^910^ inhibits the interaction of FAK with paxillin, then regulate of the FAK-paxillin complex and it is possible that ERK-modulated disassembly of the FAK-paxillin complex is involved in focal adhesion disassembly [48]. This emphasizes that ERK is an important factor in the regulation of cell migration.

It is unknown how silence of Tβ10 increases cell migration and metastasis of CCA. However, it is possible that suppression of Tβ10 increases the free form of G-actin, which is available for the dynamic actin polymerization especially in the cell front, thus enhances cell migration and tumor metastasis. Furthermore, Tβ10 is a key factor that interacts with Ras and inhibits Ras-dependent ERK1/2 signaling pathway [24]. It is recently reported that ERK1/2 activation mediates the expression of EGR1, which subsequently increases the invasive capability of ovarian cancer cells [49]. EGR1 also activates expression of Snail [50], a key inducer of epithelial-mesenchymal transition (EMT), which plays an important role in cancer metastasis [51-54]. In our current study, we demonstrate that Tβ10 silence-induced cell migration and metastasis of CCA may also involve ERK12, EGR1 and Snail pathways. Silence of Tβ10 substantially activated ERK1/2, and increased mRNA and protein levels of Snail and mRNA levels of EGR1 in CCA cell lines. However, silence of Tβ10 did not increase protein levels of EGR1. It is possible that Snail binds to the EGR1 promoter and represses EGR1 transcription, as well as its own promoter, thereby establishing a negative regulatory feedback loop [50,55,56].

In addition, activation of ERK1/2 can be caused by KRAS mutation in many cancer types [28]. Our data also confirm this possibility in CCA. The Ras-GTPase inhibitor, FPT inhibitor III, effectively blocked the activation of ERK1/2 and the expression of Snail as well as the wound healing rate in Tβ10-silenced CCA cell lines (M055-sh-Tβ10 and M214-sh-Tβ10).

Furthermore, high expression levels and activities of MMPs contribute to the invasiveness and metastasis potential in many types of cancers [29]. In the current study, we determined the relationship between silence of Tβ10 and expression of MMPs in CCA cell lines. Our data showed that stable Tβ10 knockdown cells (M055-sh-Tβ10 and M214-sh-Tβ10) had a relatively higher expression of MMP3, MMP7 and MMP9 than their control cells. The loss of Tβ10 in CCA may have a causal relationship with the increased expression of MMPs, which may enhance CCA metastasis.

Currently, functional roles and regulation mechanisms of Ras, ERK1/2, EGR1, Snail and MMPs in CCA metastasis are not fully understood. Further investigation into the whole picture of signaling mechanisms and protein interactions mediated by Tβ10 is warranted. It is not clear whether the current findings obtained from the research in the fluke-associated CCA are applicable to other types of CCA with different etiology. Now, there are no reports on the relationship between Tβ10 and other types of CCA. It could be a great opportunity for future investigation.

## Conclusions

The present study demonstrates that Tβ10 expression is relatively high in the primary CCA tumor tissues; while it is dramatically reduced in the metastatic tumors. Overexpression of Tβ10 reduces cell migration; whereas silence of Tβ10 expression enhances CCA cell migration and invasion *in vitro*. Loss of Tβ10 expression accelerates tumor metastasis of CCA in the nude mouse model. Silence of Tβ10 mediates migration of CCA cells possibly through the activation of Ras, ERK1/2 and upregulation of Snail and MMPs. More studies in the molecular mechanisms of Tβ10 associated with cell migration and metastasis in CCA are warranted in order to develop new strategies to treat CCA.

## Competing interests

The authors declare that they have no competing interests.

## Authors’ contributions

SS, KS, RK, CW, KV, SO, QY, SW and CC designed research; SS and SO performed research; RK performed in-house PCR array; SS, KS, RK, CW, KV, SO, QY, SW and CC analyzed data; and SS, SW and CC wrote the paper. All authors read and approved the final manuscript.

## Pre-publication history

The pre-publication history for this paper can be accessed here:

http://www.biomedcentral.com/1471-2407/13/430/prepub
